# Dr. Auchincloss on Injuries of the Head

**Published:** 1830-07-01

**Authors:** 


### CLINICAL REVIEW.

### XXXI.

### GLASGOW ROYAL INFIRMARY.

### INJURIES OF THE HEAD.*

Dr. Auchincloss has published a quarterly report of the cases under his care at the above institution, with the clinical remarks which they elicited, in the last number of our valuable cotemporary of Glasgow. From the many interesting cases in which the report abounds we shall select the subject of injuries of the head for the present article. It is a subject which is far from exhausted, although so much has been written on it at various times by some of the ablest surgeons that have adorned the profession in this country. The truth is, that cases of injury of the head are almost infinitely varied, and nothing but immense experience, or a long and careful collation of really *authentic* facts can ever lead to an extended and satisfactory acquaintance with the general bearings and more particular details of this class of accidents. We would recommend gentlemen to peruse the paper of Mr. Brodie in a late number of the Medico-Chirurgical Transactions. It is one of unassuming but genuine merit, and it is no small recommendation to the surgical reader, that he may rely implicitly on the facts and the statements it contains. This is unfortunately not the case with some of the more showy and tinselled productions of the day. With these few observations we pass to the report before us.

* Glasgow Journal, No. X.

CASE 1. *Concussion—Symptoms of partial Pressure.* "James Armour, aged 27, a quarrier—admitted 8th May. The accident had happened a week previously, and was occasioned by a quantity of earth falling upon him. He remained insensible for two hours, during which considerable hæmorrhage is said to have taken place from the right ear. On recovering, he felt acute pain on the right side of the head, for which he was twice bled with marked relief. On admission, he had little or no uneasiness, and complained chiefly of giddiness when he attempted to raise his head from the pillow. He was perfectly blind of the left eye, the pupil of which, however, contracted freely on exposure to light. The sense of hearing of the right ear was much impaired. His mouth was slightly drawn to the left side, which deformity increased greatly when he spoke. The pulse was about 60, the skin cool, and in other respects he was under no fever. The bowels were freely opened, and, on the following day, a large blister was applied to the head. On the 13th, four days after admission, there was an accession of feverish symptoms, with return of headach and deep-seated pain under the right ear, the hearing of which was completely gone. The pulse was full and had risen to 90. He was bled from the arm, and the blistered surface was ordered to be dressed with savine ointment. On the 14th, the pain of the head and ear had all but gone, and in every other respect he felt greatly better. The features were much less distorted, and he had recovered in part the hearing of the right ear. The pulse had fallen to 70. The blister was repeated to the head, and he was ordered gr. ii. calomel three times a day. In the course of a week his mouth became affected, and the medicine was therefore omitted. He continued daily to improve, and was dimissed well on the 8th June, having been a patient in the hospital exactly a month. At this date, the features did not appear at all to be distorted, except when he laughed or attempted to smile. He did not recover the sight of the right eye."

The case is not drawn up with much care, for in one place it is said that the vision of the *right* eye was destroyed, and in another, of the *left*. Such carelessnesses as these are always reprehensible. Dr. Auchincloss observes, and every experienced surgeon will agree with him, that the case was evidently one of concussion with partial pressure from extravasation. The recovery from the state of insensibility and the continuance of partial palsy of the face with hæmorrhage from the ear are sufficient evidence on these points.

"This person was not admitted till after the eighth day, by which time the inflammatory stage subsequent to the accident, had subsided under the usual means. The principle observed in the treatment was to excite absorption, if the symptoms depended on effusion, or, supposing these to be the consequence of concussion, to rouse the tone of the nerves. Both ends, probably, were accomplished by the measures had recourse to;—namely, counter-irritation, and the exhibition of mercury.

"On the fourth day from admission, there was a return of feverish symptoms, with pain in the situation of the ear, which readily yielded to a full bleeding. This perhaps depended on an inflammatory state of the brain, excited by the effused blood. In cases of effusion into the substance or on the surface of the brain, we often meet with accessions of inflammation, occurring in the progress of its absorption. In this respect, the presence of the fluid seems to act merely as a cause of irritation, and may therefore be compared in its operation to that of an exostosis, or any other tumour, in producing fits of apoplexy, &c.

"One other circumstance in this case rather uncommon, may be mentioned, viz.—that the motions of the iris of the blind eye remained perfectly free. From this it is evident that the mobility or immobility of the iris, is by no means an index of the sensibility of the retina, at all to be depended on. This is owing to the iris being supplied by the ciliary nerves, which proceed from the lenticular ganglion. I have frequently met with cases of amaurosis with active pupils, and *vice versa*,—an immoveable state of the pupil with perfect sensibility of the retina."

CASE 2. *Concussion—Slight Compression—Secondary Symptoms.* James Gorman, æt. 30, admitted June 4th, in a state of insensibility, with abrasion of the left cheek and dislocation of the left thumb. When placed in bed he lay in a semi-comatose condition, returning no answer when loudly questioned, moaning frequently, and restlessly tossing his limbs when pinched. Pulse 120, sharp; respiration slow; skin hot and dry. On the day preceding his admission he had leapt from the roof of a one-story house, whilst intoxicated, and pitched upon his head. *Venesection to twenty ounces—sixteen leeches to the head, with cold lotions—purge of calomel and senna draught.* The bleeding was repeated on the 5th, and on the 6th he was more composed, had a pulse at 90, and put out his tongue when requested; the pupils, especially the right, were dilated. On the 8th he was quite sensible, but complained of slight head-ache; pulse 70; tongue clean and moist. That night was restless, and on the 9th he had a vacant expression of countenance, with stupidity in answering questions. *Blister to the head.* In the night he was so delirious as to require a strait-jacket, but it was delirium without pyrexia. *Blistered surface dressed with savine—calomel* gr. ij. *3tiis horis—low diet.* The mouth became "smartly sore," the patient got daily better, and was dismissed cured on the 1st July.

In a fortnight he was re-admitted with vertigo, double vision on walking, and occasional pain shooting through the head. *Cupped on the nucha, blis-*

*tered, and ordered calomel pill thrice daily.* In five days the mouth became affected and all the symptoms were much relieved. On the 29th the medicine was re-commenced, and a caustic issue established in the neck; violent ptyalism ensued, the symptoms wholly disappeared, and the patient was again dismissed on the 10th of August. On the 23d he was again received with nearly the same symptoms as before, but little or no pain in the head. The issue was re-opened, the mercury repeated, and a strong liniment applied to the head. The mouth becoming affected, the symptoms "began to wear off;" the mercury was repeated, and the patient left the hospital on the 15th September. He shewed himself again in February on his return from Ireland, and stated that he continued to enjoy good health.

This is an interesting case, and in all probability slight extravasation or even laceration of the brain was joined with the concussion. The occurrence of what we have termed "secondary symptoms," and their *apparent* subjection to mercury, are not amongst the least important features. We say apparent subjection to the influence of calomel, for the evidence of its powers is not quite positive in the written details of the case. Let us hear the opinions of Dr. Auchincloss.

"The treatment was very simple. The man had suffered a severe mechanical shock, to which inflammation, as a necessary consequence, had succeeded. The chief indication then was to subdue arterial action. This was effected by bloodletting in sufficient quantities to control the force of the circulation, the advantages of which were strikingly apparent. Although rather a tedious case, it was otherwise very interesting. The frequent return of vertigo, double vision, &c. proves to me that it was in every likelihood complicated from the commencement with partial laceration of the brain, and slight extravasation of blood; or that perhaps effusion of serous fluid had happened after the cessation of the inflammation. I should imagine it more likely to have been caused by the first. One or other of these circumstances, however, sufficiently explains the loss of memory, giddiness, double vision, &c. We often find similar effects to supervene on the cure of phrenitis, or in cases of fever where there has been a great flow of blood to the head, and more particularly in injuries of the head with decided extravasation of blood.

"For the removal of these symptoms, the treatment consisted in obviating, in the first instance, the chance of the recurrence of inflammation, and afterwards in favouring absorption by counter-irritation. The former was accomplished by cupping and the use of purgatives; and the latter by the repeated application of blisters, and the formation of a caustic issue. After a sufficient trial, these were found to be perfectly ineffectual. Mercury as an ulterior measure was therefore had recourse to, and used freely and repeatedly with the best effect.

"As soon as the mouth got fairly under the influence of the medicine, the unfavourable symptoms uniformly began to subside. Their frequent recurrence convinces me that the brain perhaps may have been slightly lacerated, giving rise to partial effusion of blood, either at the time of the accident, or at a period subsequently to it. Instances of secondary hæmorrhage are certainly rare, though several are on record. The same line of treatment is applicable to both cases."

CASE 3. *Concussion—Scalp Wound—Secondary Symptoms.* John Curbans, æt. 21, a slater, admitted Sept. 8th, with a wound an inch long through the integuments of the right eye-brow, and another over the right temple where the bone was exposed, and the probe could be passed for three inches upwards and backwards. He had little head-ache, but chiefly complained of a fixed pain in the hypogastrium with inability to make water, and a quick pulse. He had fallen from a scaffold two stories high on the preceding day, struck his head in the descent against the edge of a wall, and received a quantity of slates upon his back. *Catheterism—edges of wound brought together by plaster—fomentations to the belly—castor oil.* The fever being increased next day, he was bled to xvi. ozs. and had twelve leeches to the belly with relief. The bleeding was repeated on the 10th, and saline mixture ordered, but on the 12th there was a recurrence of pain in the belly with increase of pyrexia, for which he was cupped on the loins to 12 ozs. From this time he no longer required the catheter. On the 21st he had a rigor, the wound looked pale, and the discharge which had previously been healthy was scanty and thin, he had malaise and tightness about the head, the countenance was flushed, and the pulse 112. *Bled from temporal artery —blister to nape of neck—castor oil—cal.* gr. ij. *ter die.* He was immediately relieved by the bleeding, and on the 22d had no pain of head. *Calomel continued—blistered surface dressed with resin ointment.* On the 26th, the mouth was sore, and the calomel discontinued; he felt well, and the discharge was good.

On the forenoon of the 27th he had another rigor, and the wound above the temple had a glazed appearance with copious discharge of brown serous fluid. On introducing the probe it passed nearly four inches backwards to an elastic fluctuating swelling; this was immediately laid open to the extent of two inches and a half, exposing a considerable surface of the bone bare and rough. Eight ounces of blood were lost with relief to the pain in the head; pulse 116, sharp—countenance flushed—tongue clean—mouth nearly free from mercurial fetor. *Dose of physic—blister to the head.* On the 28th he was bled to ten ounces, and the calomel recommenced every three hours. On the 30th the pain of the head was gone, the pulse was 84, calm, the wound clean, the mouth slightly touched. Ptyalism took place, the mercury was omitted on the 3d October, the wound granulated and healed, on the 26th the patient was dismissed, and he has remained quite well to the present time.

The issue of this case is extremely favourable in comparison with what frequently follows the development of the symptoms it presented. Rigors on the 14th and subsequent days, are but too often the overture to the fatal drama of pus on the meninges of the brain, or purulent depôts in the liver or cellular membrane. Calomel was used in this, as the preceding case, with a boldness not commonly evinced in the treatment of these cases. It will require further experience to determine its merits or demerits. Dr. Auchincloss' clinical remarks are brief and to the purpose.

"The preceding is an instance of concussion both of the spine and brain. Although the fall seemed to have been very severe, yet he suffered but momentary stupor, followed by vomiting. Symptoms of cerebral disturbance did not evince themselves till 14 days subsequent to the accident.

"These, as also the unfavourable symptoms which took place on the 27th, when he had a relapse, were ushered in, as is usually the case after injuries of the head, by a rigor, with a glazed, unhealthy appearance of the wound. When admitted he was free from fever, the local symptoms being pain at the lower part of the back, and in the hypogastrium. There was also retention of urine, with paralysis of the sphincter ani. These subsided after free depletion, both general and local, fomentations, purgatives, and the use of the catheter. Palsy of the bladder is a common effect of injuries of the back, but, in this instance, it seemed to be accompanied by actual inflammation of that organ, or of parts in the immediate vicinity. The treatment at first had recourse to was, doubtless, beneficial in lessening the tendency to inflammation of the brain, having lost during that period upwards of forty ounces of blood, so that he was very much reduced by the time the bad symptoms appeared.

"The utility of mercury after bloodletting was well marked in this case. The unpleasant symptoms disappeared, almost immediately, on the mouth becoming decidedly under the influence of that medicine. At both relapses, venesection was not sufficient to remove the pain, &c. Mercury, therefore, may be considered as one of the best auxiliaries to bleeding in the treatment of such cases. To ensure its full effect, however, I am persuaded that the medicine ought to be carried to the extent of producing smart ptyalism, as is so beautifully exemplified in the cure of iritis, and of some of the other disorganizing inflammations of the eyeball. In addition to its other effects of exciting absorption, &c. it may not improperly be said to act beneficially, partly, as a counter-irritant.

"The opinion was long entertained that a bone, when deprived of its periosteal covering, must necessarily exfoliate. The contrary, as I have often witnessed on other occasions, was seen to take place. The surface of the bone granulated, and in process of cure these granulations readily inosculated with those from the surrounding soft parts."

Case 4. *Slight Concussion—Simple Fracture of the Cranium—Severe pain in the Head.* "George M'Donald, aged 40, was admitted 27th Feb. under the care of my predecessor, Dr. Couper. On the 2d Jan. preceding, when intoxicated, he had fallen down a stair, and pitched on his head. He remained insensible for 10 minutes after the accident. With the exception of a slight abrasion of the left cheek, there was elsewhere no mark of injury to be seen. He continued at his employment during the first week. He had then a rigor, was sick and drowsy, and affected with severe shooting pain in the forehead and across the temples. At length a small puffy tumour appeared over the upper part of the left parietal bone. This, having extended, was opened a few days previous to admission, and a quantity of matter discharged. In this situation, the bone was found broken into several small pieces, and slightly depressed. The integuments were undermined, and the probe grated on rough bone to the extent of two inches around the opening. There was no paralysis, and he seemed perfectly intelligent. Pulse 80. Bowels slow. He was bled from the arm, had leeches applied to the head, and was purged with decided relief. The headach again became severe on the 4th March, when leeches were repeated. He remained in the hospital till the 21st April, during which period he was never free of head-ach. The treatment had recourse to was the application of leeches and cold lotions to the head, the occasional use of purgatives, and an alterative course of mercury. On his leaving the hospital, the following report is entered in the Journal: 'Has little pain in the head, and otherwise appears in good health. No portion of the denuded and depressed bone has yet come away.'

"This person was re-admitted on the 16th May. The head-ach, which had been more or less constant from the time he left the hospital, was now particularly severe. There was no other bad symptom. He was addicted to dissipated habits, and had been living rather irregularly. The sore on the upper part of the skull was about the size of a sixpence. The surrounding scalp was quite adherent. The depressed bone felt rough, and was covered with flabby granulations. The treatment consisted in the application of blisters and the use of mercury to ptyalism. He left the hospital on the 11th June, still affected with headach, though much less severe. No change had taken place on the sore.

"He was again admitted on the 4th July, and in the interval had been living very abstemiously. The pain was constant and so severe, that he could neither walk nor stoop. He complained of loss of memory. A caustic issue was inserted in the nape of the neck;—and the edges of the sore on the head, which were inverted, were pared. Two days subsequent to this, erysipelas of the scalp and face took place, preceded by a rigor. Delirium ensued early, and he remained very ill for nearly a week. The affection having subsided, healthy granulations sprung up from the surface of the sore, which speedily cicatrised, no exfoliation of bone taking place. He left the hospital perfectly free of headach on the 9th of August. I have not heard of him since the middle of Sept. at which date he was well and able to follow his usual employment—a tanner."

Dr. Auchincloss makes no remark on this case, but if the pain should return and continue severe, it will probably be necessary to trephine the depressed and diseased bone. Such was the successful proceeding adopted in some of the cases detailed by Sir Everard Home. By the way we believe that Mr. Brodie is preparing a memoir on the secondary consequences of injuries of the head.

The next and last case that we can notice, is brought forward to shew that the "puffy tumour" of Mr. Pott does not necessarily accompany the formation of matter on the dura mater. The case is short and incapable of abbreviation.

"A gentleman, aged 21, in descending a stair, fell forwards and pitched his head against the wall at the bottom of the stair. He was slightly stunned by the blow, and after a few minutes, rose up and walked home. There was no visible mark of injury on the head. He continued in his usual state of health for nearly a month. At length he became affected with head-ache, vertigo, and other cephalic symptoms. The pulse was about 40. Stupor and convulsions ensued, and he died apoplectic seven weeks from the receipt of the accident.

"*Inspection.* On reflecting the scalp and pericranium, which was everywhere firmly adherent to the skull, two fissured fractures were discovered on the upper part of the left parietal bone. These were situated an inch apart, and ran parallel to each other in an oblique direction, towards the sagittal suture, where they terminated. The dura mater underneath was separated from the bone, which was rough to the extent of three square inches. This space was occupied by dark coloured sanies, with a sloughy state of the outer layer of the dura mater. The inner surface of this membrane was of its natural smoothness and colour. The brain, particularly the cerebellum, was unusually soft."

We shall notice the other topics embraced in this report at another opportunity. Dr. Auchincloss is a well-informed, judicious, and apparently accurate surgeon. These are no mean recommendations in the present days of ostentatious pretension, and unblushing contempt of truth in medical periodical literature.

---

